# Morphological changes on the human liver during minimally invasive surgery: Implications for image-guided interventions and surgical navigation

**DOI:** 10.1007/s00464-025-12392-y

**Published:** 2025-11-23

**Authors:** Varatharajan Nainamalai, Håvard Bjørke Jenssen, Luca Boretto, Nikhil Pramod Kumar, Andreas Westenvik Espinoza, Egidijus Pelanis, Bård I. Røsok, Ole Jakob Elle, Ingrid Schrøder Hansen, Bjørn Edwin, Åsmund Avdem Fretland

**Affiliations:** 1 The Intervention Center, Rikshospitalet, Oslo University Hospital, 0372 Oslo, Norway; 2 Division of Radiology and Nuclear Medicine, Oslo University Hospital, 0372 Oslo, Norway; 3 Department of Hepato-Pancreatic-Biliary Surgery, Oslo University Hospital, 0372 Oslo, Norway; 4 Department of Informatics, University of Oslo, Problemveien 11, 0313 Oslo, Norway

**Keywords:** Computed tomography images, Segmentation, Image registration, Pneumoperitoneum

## Abstract

**Background:**

Minimally invasive liver resection and ablation depend on surgical planning and image guidance. Surgical planning is normally based on preoperative imaging. The position, shape, and volume of the abdominal organs change during laparoscopy, which challenges image registration and reduces surgical precision. This study aims to analyze the morphological changes of the liver and spleen from pre- to intraoperative (with pneumoperitoneum) computed tomography (CT) images.

**Methods:**

We used portal venous phase pre- and intraoperative CT images from 15 patients who underwent laparoscopic liver ablation in general anesthesia under 12 mmHg pneumoperitoneum at Rikshospitalet, Oslo University Hospital, Oslo, Norway. A rigid registration, based on spinal landmarks, was used to register intraoperative to preoperative CT images. Morphological features were extracted and statistically analyzed for the liver and spleen.

**Results:**

The liver volume decreased by 12% from the preoperative to the intraoperative CT scan. The mean cranial movement of the liver was 45 mm between pre- and intraoperative CT images. A few morphological radiomic features changed significantly for both liver and spleen.

**Conclusion:**

To the best of our knowledge, this is the first published study in humans to analyze the morphological changes of the liver and spleen during pneumoperitoneum. The results show a significant reduction in liver volume and change in shape and position of the liver during such laparoscopy. This deformation from preoperative to intraoperative imaging poses significant challenges for image registration, which is crucial for surgical navigation. These findings highlight the need for updated intraoperative navigation using imaging and registration to ensure accurate surgical planning.

**Supplementary Information:**

The online version contains supplementary material available at 10.1007/s00464-025-12392-y.

Navigation systems for laparoscopic surgery or ablation usually depend on 3D models derived from preoperative images and registered intraoperatively to provide guidance. In abdominal laparoscopic procedures, the abdomen is usually insufflated (pneumoperitoneum) to 12 mmHg using carbon dioxide (CO_2_) gas to generate enough space to utilize the surgical instruments inside the abdomen. The pneumoperitoneum alters the position and the morphology of the internal soft tissues. Therefore, even if the preoperative images or models are rigidly registered to the intraoperative ones using landmarks on soft organs, such as the liver or spleen, they may not align with the corresponding landmarks in the pre-operative imaging data [1].

Organ motion due to ventilation, during intraoperative anesthesia, also affects registration accuracy. Many challenges exist due to the dynamic deformation of organs during surgery. Hence, preoperative planning/models cannot accurately represent intraoperative anatomical changes [2]. Pneumoperitoneum during abdominal surgery can cause significant hemodynamic, ventilatory, and cardiovascular complications. These risks can be avoided by clearly understanding the effects of laparoscopic surgery and implementing a well-defined surgical plan developed collaboratively by a multidisciplinary team, including anesthesiologists and surgeons [3].

Respiratory phases during surgery and pneumoperitoneum can also cause liver volume changes [4]. Zhang et al. [4] studied (porcine) intraoperative changes due to pneumoperitoneum in terms of surface area and volume of the liver, the vascular diameter of the aortic lumen, inferior vena cava lumen, and portal vein lumen. They observed that 13 mmHg pressure caused significant changes in liver surface area, volume, and the diameter of blood vessels. Other studies have shown that inspiration and pneumoperitoneum in porcine models caused significant changes in liver position and peripheral deformation [5, 6].

Intraoperative imaging could be useful for correcting anatomical shifts during the guidance of laparoscopic liver procedures [5, 7]. Kenngott et al. [8] studied the effects of the respiratory phase, pneumoperitoneum for laparoscopy, and laparotomy on liver volume in a live porcine model. The authors observed that significant changes in liver volume were associated with pneumoperitoneum, laparotomy, and respiration.

A previous meta-analysis assesses the effects of high- versus low-pressure carbon dioxide pneumoperitoneum (CDP) and the varying duration of CDP in patients during abdominal surgery [9]. Postoperative liver function markers, including ALT (Alanine Transaminase), AST (Aspartate Aminotransferase), and TB, were assessed on postoperative days 1, 3, and 7 in the study groups. The study suggests that the duration of CDP during laparoscopic abdominal surgery may be associated with liver injury. However, studies comparing laparoscopic and open approaches for colorectal cancer resection and gastric bypass have yielded inconsistent results. Other studies on low intra-abdominal pressure have shown reduced postoperative pain, shorter hospital stay, less increased liver enzymes, etc. [10–17].

Understanding the effect of pneumoperitoneum during laparoscopy compared with the preoperative images is a basis for efficient image registration, surgical planning, and navigation. There are a few experimental studies that exist in the literature on porcine models [4–8]. However, to the best of our knowledge, there are no studies on changes of organ morphology between pre- and intraoperative (with pneumoperitoneum) CT images in humans. In this work, we study the effect of pneumoperitoneum and general anesthesia on the liver, spleen, and liver vessels using pre- and intraoperative computed tomography image segmentations of human data.

## Materials and methods

### Pre- and intraoperative computed tomography images in laparoscopic ablation

The pre- and intraoperative computed tomography (CT) images and electronic health records of a cohort of patients who underwent laparoscopic ablation were collected from Rikshospitalet, Oslo University Hospital, Oslo, Norway. A total of 15 patients were included, all were Caucasians, 12 males and 3 females. The mean age at the time of laparoscopic ablation was 67 years. The mean body mass index (BMI) was 26. Based on examinations by a consultant abdominal radiologist (no elastography information available), among the 15 patients, 6 had visible steatosis, 7 had a normal liver appearance, and 2 had cirrhosis.

Intraoperative CT images were acquired during laparoscopic ablation with an intraperitoneal pressure of 12 mmHg. Preoperative CT images were acquired up to 5 weeks before the procedure. Both the pre- and intraoperative CTs were contrast-enhanced portal venous phase. Intraoperative CTs were acquired during the expiratory phase.

The preoperative images were acquired using CT scanners from Siemens Healthineers (SOMATOM Definition Flash, SOMATOM Force, SOMATOM Edge Plus), Toshiba (Aquilion PRIME, Aquilion ONE), GE Medical Systems (Revolution CT, LightSpeed VCT), and Philips (Ingenuity CT, Ingenuity Core 128). All the intraoperative CTs were acquired using a sliding gantry SOMATOM Definition Edge 128 slice, Siemens Healthineers. The kilovoltage peak (kVp) used in the preoperative CT scan was 80, or 100, or 120, while in intraoperative CT scan was 70, or 80, or 100.

**Fig. 1:**
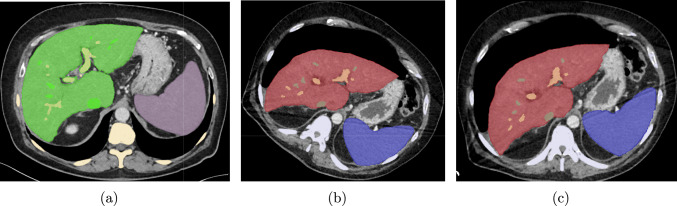
Axial slice of a **a** preoperative supine position CT image overlaid segmented labels (Green-liver, violet-spleen, yellow-spine, thick green-hepatic vein, and thick yellow-portal vein), **b** Intraoperative tilted position CT image with pneumoperitoneum overlaid liver parenchyma (red), spleen (blue), spine (gray), and hepatic vein (green), and portal vein (orange) segmentations, and **c** (Registered using spinal point) Transformed and resampled intraoperative CT image and its segmentations

Pre- and intraoperative CTs were acquired on different days; therefore, the patient positioning and respiratory phase are different. Furthermore, CT volume dimensions and spacing may differ between pre- and intraoperative CT images. Therefore, image shown in Fig. 1(b) does not correspond to the same slice as the preoperative image shown in Fig. 1(a).

### Image processing and criteria for segmentations

Image dimensions and image spacing of preoperative CT images varied across patients as (512 − 768) × (512 − 768) × (73 − 338) and (0*.*48 − 0*.*98) × (0*.*48 − 0*.*98) × (2 − 3) mm, respectively. Similarly, the image dimensions and image spacing of intraoperative CT images were 512 × 512 × (100 − 312) and (0*.*53 − 0*.*98) × (0*.*53 − 0*.*98) × 2 mm, respectively.

The liver parenchyma, liver vessels, spleen, and spine segmentation from pre- and intraoperative (with pneumoperitoneum) CT images were performed. A radiologist (HBJ) with eight years of abdominal imaging experience manually corrected the deep learning-derived liver parenchyma, liver vessels, and spleens using the 3D Slicer. Finally, two surgeons (AF and BE) manually evaluated all the segmentations using 3D Slicer.

The spine in pre- and intraoperative CT images was segmented using a threshold of 120 as the lower Hounsfield units in 3D Slicer [18]. A variant of the 3D UNet deep learning model for liver parenchyma and liver vessels was trained using the Oslo-COMET dataset [19–23] and inferred pre- and intraoperative CT images.

Liver parenchyma segmentation excluded the extrahepatic portal vein and the gallbladder. The inferior vena cava (IVC) was segmented starting from the first point of contact with the liver parenchyma on the inferior aspect and extended cranially until the last slice showing liver parenchyma or until the vessel merged with the left atrium. The portal vein was segmented from the confluence of the splenic vein and superior mesenteric vein.

### Registration of intra to preoperative CT images

All preoperative CT images were acquired in the supine position. During laparoscopy, eight patients were scanned in the supine position, and seven were scanned in a semi-oblique position (30–45 degrees right side upward). For patients in the semi-oblique position, measurements of the organ displacement and deformation, registration of pre- and intraoperative CT images were necessary. Thus, both the breathing stage and patient position would be different from pre- to intraoperative images. In order to measure the shape changes, registration of pre- and intraoperative CT images is necessary.

Registration was performed by manually selecting four landmarks on the spinous processes of TH12 and L1 in the superior articular process in the clockwise direction on both pre- and intraoperative CT images. We performed point-based rigid registration based on the selected four landmarks on the spinous processes positions.

### Assessment of morphological changes in liver and spleen

To analyze the morphological impact of pneumoperitoneum on the liver and spleen, shape features of the respective organs were calculated in pre- and intraoperative CT image segmentation. The 3D shape features were extracted using the PyRadiomics package (version 3.1.0) [24]. Volume changes in normal and pathological livers were compared with an unpaired two-sample Levene’s Test for Welch’s *t* test, which accounts for unequal variances (Levene’s test, *p* < 0*.*027).

Morphological features and registration method have been briefly described in Supplementary Material Sect. 1. Theoretical explanations for the morphological features are listed in Supplementary Material Table 1. The complete morphological shape features of pre- and intraoperative liver and spleen data are provided in Supplementary Material Tables 2-5

### Cranial-caudal liver displacement

The cranial-caudal displacement between the first liver slice of the pre- and registered intraoperative CT images was measured about the spine. For this, we segmented the whole axial slice of the CT image where the liver is visible in the axial direction of the pre- and resampled intraoperative CT. The liver displacement between the pre- and intraoperative CT images was measured by counting the number of slices between the two segmented slices and presented in Supplementary Material Table 6.

### Statistical analysis

The Shapiro–Wilk test was used to check the normal distribution of the differences between morphological features of the liver/spleen from pre- to resampled intraoperative CT images. If the differences follow normal distribution, a paired *t* test was used with the null hypothesis (*H*_0_) that the mean difference between paired samples is zero. In case of skewed data, the Wilcoxon Signed-Rank test was used to test the *H*_0_ that the median difference between paired samples is zero. The alternative hypothesis (*H*_1_) was considered that there is a significant difference between the features of pre- and insufflated intraoperative CT liver/spleen shape features. We considered the level of significance *α* = 0*.*05 in all statistical tests, and the statistical analyses were performed using SciPy with Python packages. A detailed description of statistical analysis is provided in Table 3.

## Results

### Registration of tilted intra to preoperative CT images

The error between pre- and intraoperative CT registration with respect to each point is given as Point:1-Point:4 and shown in Table 1. The average registration error for each patient, the average of all four points of the same patient, is provided as RMean. We also calculated the angle of deviation and the translation between pre- and intraoperative CT images.

**Table 1:** The registration error between pre- and intraoperative CTs using spinal landmarks, deviation angles in each axis, and translation of the intraoperative CTs with Pneumoperitoneum

Error (mm)						Deviation Angles			Translation		
Case	Point:1	Point:2	Point:3	Point:4	RMean	Roll	Pitch	Yaw	*T*_*x*_	*T*_*y*_	*T*_*z*_
1	0.57	0.8	0.77	0.46	0.649	− 1.15	0.24	23.89	− 65.25	− 168.27	2303.32
2	1.83	2.06	1.48	1.04	1.606	1.45	− 1.34	33.6	− 100.41	30.31	− 1940.1
3	0.21	0.54	0.99	0.78	0.63	1.37	4.02	28.56	− 66.4	32.73	− 314.47
4	1.55	1.63	0.97	0.86	1.254	1.66	1.09	1.13	24.6	− 162.67	383.61
5	1.43	0.45	1.37	1.9	1.288	− 6.28	3.19	2.71	35.96	− 285.81	1412.7
6	0.65	0.88	0.81	0.48	0.702	2.88	4.8	28.11	− 48.58	− 256.7	2485.82
7	3.99	4.39	3.05	2.36	3.447	4.4	0.82	23.21	− 77.66	34.31	1705.42
8	1.02	1.52	1.63	0.83	1.252	− 1.22	3.02	3.89	14.1	53.43	729.76
9	1.51	0.91	1.15	1.65	1.306	0.85	0.66	45.98	-71.04	− 0.48	2315.25
10	1.04	0.59	1.03	1.28	0.987	2.73	1.73	3.77	13.84	32.6	1866.07
11	1.93	1.31	0.8	1.54	1.396	3.39	− 1.62	2.99	− 26.69	− 97.04	290.43
12	2.34	1.61	0.83	2.02	1.7	2.42	3.01	− 2.26	− 35.22	39.79	906.32
13	0.69	1.26	1.71	1.24	1.227	2.76	− 3.09	24.92	1.73	10.57	3922.87
14	1.07	0.86	1.02	1.22	1.044	2.71	0.24	2.32	− 0.85	− 105.49	1234.79
15	1.14	0.88	0.71	0.95	0.92	− 0.64	1.7	− 1.79	− 18	57.61	334.31
Minimum	0.21	0.45	0.71	0.46	0.63	− 6.28	− 3.09	− 2.26	− 100.41	− 285.81	− 1940.1
Maximum	3.99	4.39	3.05	2.36	3.447	4.4	4.8	45.98	35.96	57.61	3922.87
Median	1.14	0.91	1.02	1.22	1.252	1.66	1.09	3.89	− 26.69	10.57	1234.79
Mean	1.40	1.31	1.22	1.24	1.29	1.16	1.23	14.74	− 27.99	− 52.34	1175.74

Point:1 to Point:4-Chosen four points on the spines. RMean represents the average registration error from Point:1 to Point:4 for each case. Deviation angles are provided in degrees.

The tilted cases in the intraoperative CT images can be identified from the increased angle of deviation "Yaw" in Table 1. The minimum, maximum, median, and mean angles of rotations in the tilted intraoperative pneumoperitoneum patients are 23.21, 45.98, 28.11, and 29.8 degrees, respectively. Similarly, the min., max., median, and mean deviation in the supine position pre-operative patients are -2.26, 3.89, 2.52, and 1.6, respectively. Also, the translation *T*_*z*_ is significantly higher than in other directions.

**Fig. 2:**
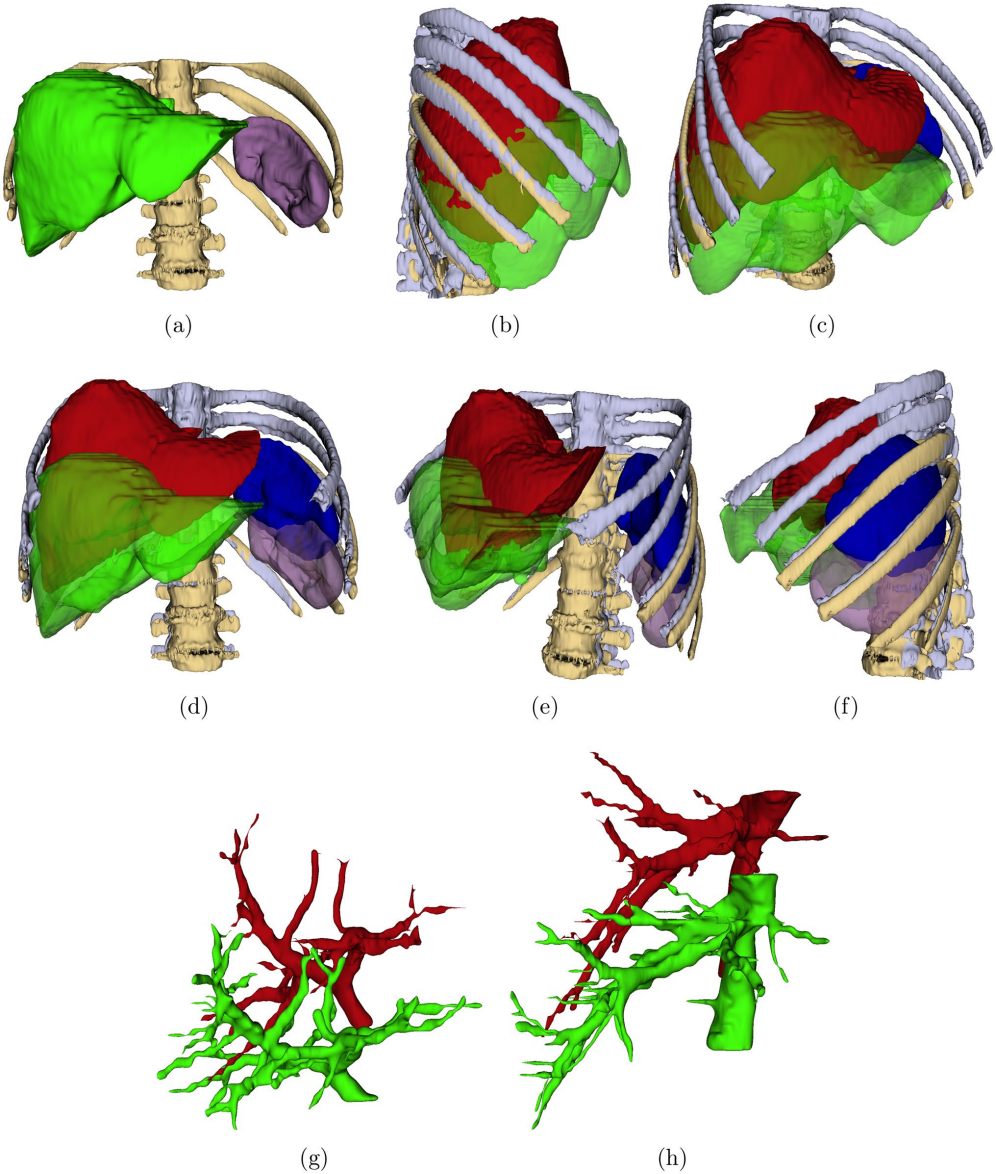
Anterior view of segmentations of **a** preoperative CT spine (yellow), liver (green), and spleen (violet), **b**–**f** different views of registered, resampled intraoperative CT with pneumoperitoneum spine segmentation (gray), liver (red), and spleen (blue) along with preoperative spine, liver, and spleen, **g** portal vein segmentations of pre (green)- and registered resampled intraoperative CT (red), and **h** hepatic vein segmentations of pre-(green) and registered resampled intraoperative CT (red)

After the rigid registration, all the intraoperative CT images and their segmentations were resampled to the pre-operative CT images using the corresponding transformations. A visual comparison of liver, spleen, portal, and hepatic veins of the pre- and registered resampled intraoperative CT image segmentations is shown in Fig. 2.

### Pneumoperitoneum effect on shape of liver

During general anesthesia and pneumoperitoneum, the liver was displaced cranially, there was a significant volume reduction, and the shape was changed. From pre- to intraoperative CT, the mean craniocaudal displacement of the liver was 45 mm (range of 12–78 mm, SD 17 mm) (Supplementary Table 6). Furthermore, a significant reduction in liver volume was observed, with a mean decrease of 210 mL (*p* = 0*.*0004). A large variability in liver and spleen volumes and deformations could be observed, likely related to liver texture/pathology.

**Fig. 3:**
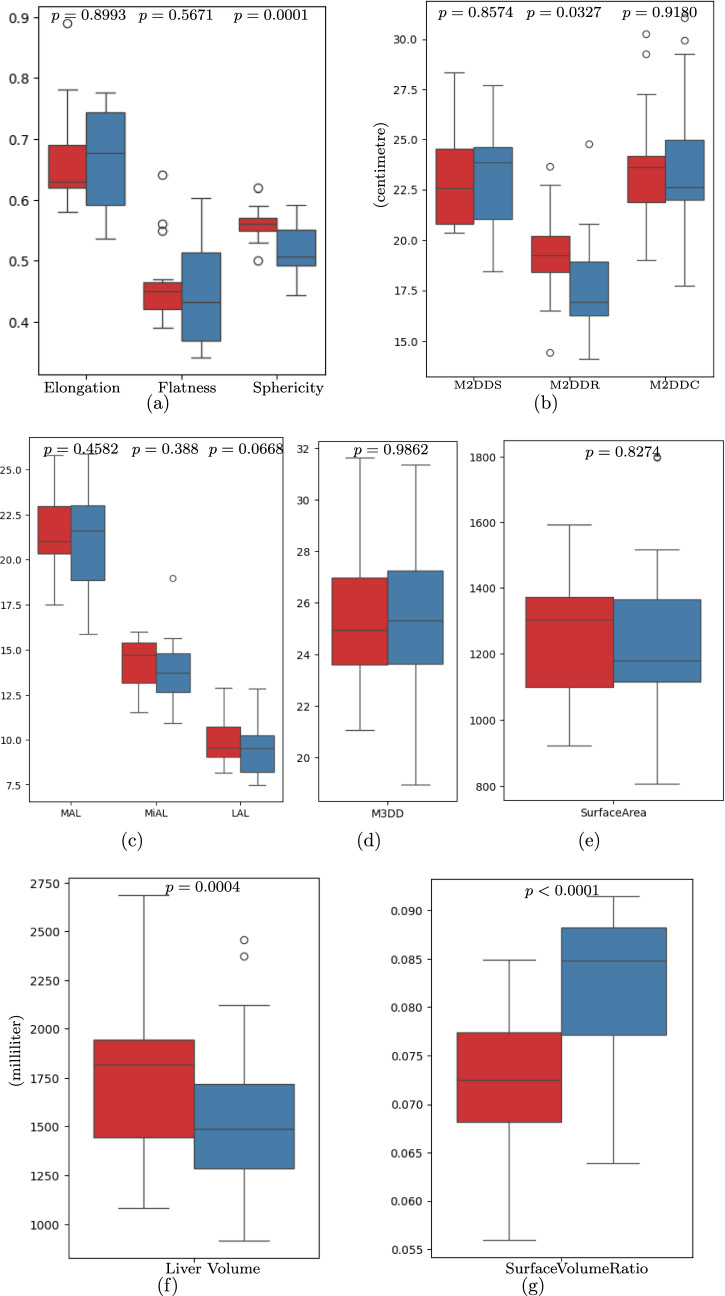
Distribution of morphological shape features variation in pre- (red) and intraoperative (blue) CT liver segmentation. *M2DDS* maximum 2D diameter slice, *M2DDR* maximum 2D diameter, *M2DDC* maximum 2D diameter column, *MAL* major axial length, *MiAL* minor axial length, *LAL* least axial length, *M3DD* maximum 3D diameter

The morphological features show a significant change between preoperative and intraoperative liver CT images. Specifically, the sphericity of the liver (which quantifies how closely the shape resembles a perfect sphere) decreased by a mean of 0.0392 (*p* = 0*.*0001). Additionally, the maximum 2D diameter row, which represents the longest diameter of the liver on the sagittal CT slice, showed a mean decrease of 1.63 cm (*p* = 0*.*0327). The surface-to-volume ratio (SVR) exhibited a mean increase of 0.0092 (*p* < 0*.*0001), and the comparison of pre- and intraoperative liver features distribution are presented in Fig. 3.

The distributions of other shape features, including elongation, flatness, maximum 3D diameter (M2DDS) slice, and column (M2DDC), major axial length (MAL), least axial length (LAL), minor axial length (MiAL), and surface area, were changed. However, the variation between these features were statistically not significant. The statistical analysis is presented in Tables 2 and 3 for the liver and spleen, respectively.

**Table 2:** Statistical comparison of morphological features of pre- and resampled intraoperative (pneumoperitoneum) CT image segmentations of liver

	Pre-operative liver			Intraoperative liver				Mean Difference	*p* value of paired *t* test
Feature	Min	Max	Mean	Min	Max	Mean			
Elongation	0.5755	0.8851	0.6659	0.5361	0.7767	0.6629		0.0031	0.8993
Flatness	0.3935	0.6374	0.4648	0.3406	0.6025	0.4524		0.0124	0.5671
Sphericity	0.4975	0.6215	0.5590	0.4437	0.5910	0.5198		0.0392	**0.0001**
Major axis length (cm)	17.47	25.80	21.53	15.87	25.89	21.15		0.38	0.4582
Minor axis length (cm)	11.53	16.01	14.25	10.92	18.96	13.90		0.35	0.3880
Least axis length (cm)	8.17	12.90	9.94	7.49	12.85	9.41		0.54	0.0668
Max. 2D Dia. slice (cm)	20.38	28.35	23.14	18.43	27.71	23.26		− 0.12	0.8574
Max. 2D Dia. row (cm)	14.42	23.65	19.30	14.10	24.79	17.67		1.63	**0.0327**
Max. 2D Dia. Column (cm)	19.00	30.26	23.73	17.72	31.05	23.77		− 0.05	0.9180
Max. 3D Dia. (cm)	21.06	31.65	25.53	18.95	31.37	25.53		− 0.01	0.9862
Volume (mL)	1083.29	2685.69	1771.05	914.40	2456.18	1560.38		210.67	**0.0004**
Surface area (cm^2^)	919.92	1593.79	1258.65	806.24	1799.15	1251.79		6.86	0.8274
Surface volume ratio	0.0560	0.0849	0.0728	0.0639	0.0915	0.0820		− 0.0092	** < 0.0001**

Cranial-Caudal displacement between pre-and intraoperative CT livers					
	Min	Max	Mean	Median	SD
	12	78	45	45	17

Bold values indicate statistical significance

*Min* minimum, *Max* maximum, *Dia* diameter. The plus and minus values in the Mean Difference column represent the decrease and increase in features between pre- and intra-livers, respectively

**Table 3:** Statistical comparison of morphological features of pre- and resampled intraoperative (pneumoperitoneum) CT image segmentations of spleen

	Pre-operative spleen			Intraoperative spleen			Mean Difference		*p* value of paired *t* test
Feature	Min	Max	Mean	Min	Max	Mean			
Elongation	0.5474	0.7746	0.6743	0.5377	0.8147	0.6696	0.0047		0.6280
Flatness	0.3202	0.5211	0.4221	0.3020	0.4874	0.4049	0.0172		**0.0040**
Sphericity	0.5757	0.7026	0.6411	0.5332	0.6726	0.6063	0.0349		** < 0.0001**
Major axis length (cm)	7.35	17.13	11.84	7.57	16.88	11.82	0.02		0.9248
Minor axis length (cm)	4.02	11.63	8.06	4.09	11.79	7.97	0.08		0.5151
Least axis length (cm)	2.74	8.20	5.07	2.67	8.23	4.84	0.23		0.0481
Max. 2D Dia. slice (cm)	6.55	18.45	11.63	5.93	17.29	11.24	0.40		**0.0284**
Max. 2D Dia. row (cm)	5.26	18.30	10.56	6.24	16.94	10.62	− 0.07		0.8861
Max. 2D Dia. column (cm)	8.07	16.65	11.68	7.14	18.04	11.94	− 0.26		0.3883
Max. 3D Dia. (cm)	8.08	19.02	12.93	8.55	18.09	13.01	− 0.08		0.7593
Volume (mL)	49.80	951.91	350.35	53.22	992.27	327.81	22.54		0.3046
Surface area (cm^2^)	96.08	705.91	350.80	102.38	839.96	360.22	− 9.43		0.5157
Surface volume ratio	0.0734	0.1929	0.1236	0.0847	0.1936	0.1318	− 0.0082		**0.0010**

Bold values indicate statistical significance

*Min* minimum, *Max* maximum, *Dia* diameter. The plus and minus values in the mean difference column represent the decrease and increase in features between pre- and intra-spleen, respectively

To understand the difference between the pathological group (cirrhosis and any stage of steatosis, *n* = 8) and the normal liver group (*n* = 7), we divided them into two groups and analyzed further. The mean liver volume in preoperative cases on pathological and normal cases is 1951.1 mL and 1565.3 mL (24.6% higher volume in pathological cases), respectively. Also, the mean liver volume in intraoperative cases on pathological and normal cases is 1770.2 mL and 1320.7 mL (34% higher volume in normal cases), respectively. The volume decrease observed from pre- to intraoperative CT was 181 mL in the pathological group and 245 mL in the normal liver group (*p* = 0*.*49).

### Pneumoperitoneum effect on shape of spleen

**Fig. 4:**
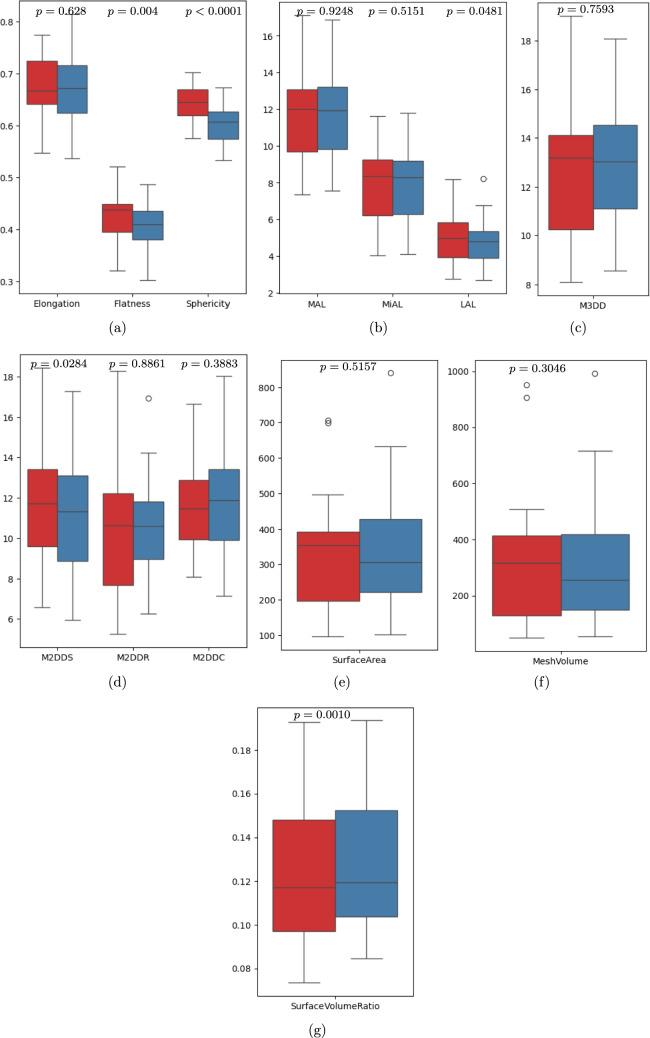
Morphological shape features variation in pre- (red) and intraoperative (blue) CT spleen segmentation. *M2DDS* maximum 2D diameter slice, *M2DDR* maximum 2D diameter row, *M2DDC* maximum 2D diameter column, *MAL* major axial length, *MiAL* minor axial length, *LAL* least axial length, *M3DD* maximum 3D diameter

In the case of the spleen, the analysis of shape features shows a significant decrease in mean flatness, sphericity, and maximum 2D diameter slice as 0.0172 (*p* = 0*.*0040), 0.0349 (*p* < 0.0001), and 0.40 cm (*p* = 0*.*0284), respectively. On the contrary, the mean difference of surface volume ratio between preoperative and intraoperative spleen increases 0.0082 (*p* = 0*.*0010), as shown in Table 3. There were no significant differences observed in the other examined shape features. However, there was a noticeable distribution shift between pre-operative and intraoperative splenic features (Fig. 4).

## Discussion

This study offers novel insights into the morphological changes of the human liver and spleen during laparoscopic procedures. The pre- and intraoperative CT images were registered using spinal landmarks. We observed that general anesthesia and a standardized insufflation pressure of 12 mmHg resulted in a 12% decrease in liver volume and a mean cranial liver displacement of 45 mm. Also, there was a difference between pathological (cirrhosis and steatosis) and normal liver mean volume reductions.

Pneumoperitoneum can affect changes in all dimensions of the liver, including volume. Therefore, we used morphological shape features from radiomic features to study liver deformation. Furthermore, significant changes in liver shape were evident when assessed through radiomic features. A decrease in sphericity shows a transformation toward a more irregular and less rounded liver shape, which can complicate the identification of anatomical landmarks. The portal and hepatic veins were compressed, showing a potential influence of pneumoperitoneum on vascular perfusion and intrahepatic pressure gradients.

Our findings are in line with previous studies of the movement of the liver and diaphragm during respiration and highlight the multifactorial nature of liver deformation [25–31]. There is an interplay of respiration, muscle relaxation, patient positioning, and intraperitoneal pressure that collectively influences organ morphology. Understanding the contribution of each factor is essential for future studies in this field.

In order to create reliable navigation systems for laparoscopic and robotic liver surgeries, dynamic imaging strategies that reflect organ positions in real-time will be essential. Future research should aim to isolate the contributions of individual factors, such as anesthesia type and respiratory mechanics, and may benefit from AI-driven models to predict organ deformation. Further integration of ultrasound as an intraoperative imaging modality may enable real-time updates of anatomical models.

As surgical techniques advance, the demand for improved visualization of patient-specific anatomy increases. Overlaying preoperative anatomical models onto target organs during surgery, preferably coupled with surgical instrument tracking, has long been a goal of navigation researchers. Emerging modalities, ranging from holograms to video overlays, have been explored in this context. The rapid evolution of robotic surgery, characterized by enhanced computational capabilities and visualization techniques, emphasizes the need for effective translation of preoperative 3D models into usable intraoperative tools. The current findings emphasize the significant challenges in achieving this goal.

General anesthesia, muscle relaxation, respiratory stage, pneumoperitoneum, patient positioning, and forces of gravity affect organ deformation. Thus, we highlight the importance and the need for advanced segmentation and registration algorithms within image-guided surgical systems, ideally implementing intraoperative imaging modalities.

This study has limitations, most importantly, a small sample size. Furthermore, it required access to a hybrid operation theater with a CT scanner, which limits the volume and reproducibility. The state of respiration was not standardized from pre- to intraoperative CT, and such standardization could reduce variation in future studies. In this study, intrahepatic changes were not analyzed separately and will be analyzed in future studies. The cohort relies on data from males (12 males, 3 females); however, this study is not focused on gender.

Also, the rigid registration technique used cannot address all anatomical changes during pneumoperitoneum. Despite all this, the study represents a foundational step in understanding the complex interactions that influence liver morphology during pneumoperitoneum. These insights pave the way for enhanced surgical planning and navigation.

## Conclusion

This study is the first to quantify changes in liver and spleen morphology due to general anesthesia and pneumoperitoneum in humans. The liver volume significantly decreased between pre- and intraoperative (with pneumoperitoneum) CT images. Our findings suggest that dynamic intraoperative imaging, combined with robust registration techniques, is essential to account for anatomical shifts during surgical guidance. Future research should aim to refine methods for modeling these dynamic changes.

## Supplementary Information

Below is the link to the electronic supplementary material.

Supplementary file1 (PDF 196 KB)
