# Supplementary material for: Morphological changes on the human liver during minimally invasive surgery: Implications for image-guided interventions and surgical navigation
Source: Surg Endosc. 2025 Nov 23;40(2):1458–68. doi: 10.1007/s00464-025-12392-y (PMC12881121; doi:10.1007/s00464-025-12392-y)
Supplement: Supplementary file 1 — Supplementary file1 (PDF 196 KB) [file 464_2025_12392_MOESM1_ESM.pdf]

1. Morphological Features, Registration Method, and Statistical Analysis

1.1. *Assessment of Morphological changes in Liver and Hepatic Veins*

In this study, radiomic features related to the three-dimensional shape of the volume of interest is considered. The shape-based features have been briefly described in Table 1. V is the mesh volume in mm^3, N_v represents the number of voxels included in the volume of interest, and V_k is the volume of a single voxel. A the surface area of the mesh in mm^2, N_f represents the number of faces (triangles) defining the Mesh, a_i, b_i, and c_i are the vertices of a single triangle of the mesh and $\mathrm{a}_i\mathrm{b}_i$ and $\mathrm{a}_i\mathrm{c}_i$ are the edges of the triangle.

1.2. *Registration of Tilted Intra to Pre-operative Images*

In general, preoperative CT images acquired in the supine position do not align with (either supine or tilted) intraoperative CT images. Hence, a rigid image registration is required to align intra- and preoperative CT images to measure the displacement and deformation. It was considered Similarity3DTransform, LandmarkBasedTransformInitializer, 2-norm, and Resample functions to register pre- and intraoperative CT images. The 3D similarity transform is mathematically described as follows:

$$T(\mathbf{x}) = s\mathbf{R}\mathbf{x} + \mathbf{t}, \tag{1}$$

where $\mathbf{x}$ is a point in 3D space, s is a scaling factor, and $\mathbf{R}$ is a 3×3 rotation matrix, and $\mathbf{t}$ is a translation vector in 3D space. The transformation in homogeneous coordinates, which is a 4×4 matrix due to the translation, can be described as

$$\begin{bmatrix} \mathbf{x}' \\ 1 \end{bmatrix} = \begin{bmatrix} s\mathbf{R} & \mathbf{t} \\ 0 & 1 \end{bmatrix} \begin{bmatrix} \mathbf{x} \\ 1 \end{bmatrix}, \tag{2}$$

where $\mathbf{x}'$ is the transformed point.

2. Results

2.1. *Morphological features*

The morphological shape features of the liver, and spleen of the pre-and registered and resampled intraoperative (transformed) CT image segmentations are shown in Tables 2, 3, 4, and 5. Notations in the Tables are El.: Elongation, Fl.: Flatness, Spl.: Sphericity, MAL: MajorAxisLength, MiAL: MinorAxisLength, LAL: LeastAxisLength, M2DDC: Maximum2DDiameter

Table 1: Morphological shape features for the liver and spleen.

Description	Definition	Formula
Voxel volume	Adding the volumes of each N_v of voxels	$V = \sum_{k=1}^{N_v} V_k$
Surface area	Sum of all the sub-surface areas of the N_f faces of the triangles.	$A = \sum_{i=1}^{N_f} A_i$, where $A_i = \frac{1}{2}\lvert \mathrm{a}_i\mathrm{b}_i \times \mathrm{a}_i\mathrm{c}_i \rvert$.
Surface-to-volume	Ratio of the measurement of total surface area and total volume.	$\frac{A}{V}$
Sphericity	Measures the roundness of the shape relative to a sphere	$\frac{\sqrt[3]{36\pi V^2}}{A}$
Max. 3D dia.	Largest pairwise Euclidean distance between the surface mesh vertices.	
Max. 2D dia. (Slice)	Largest pairwise Euclidean distance between the surface mesh vertices in the axial plane.	
Max. 2D dia. (Column)	Largest pairwise Euclidean distance between the surface mesh vertices in the coronal plane.	
Max. 2D dia. (Row)	Largest pairwise Euclidean distance between the surface mesh vertices in the sagittal plane.	
Major Axis Length	Largest axis length of the ellipsoid that encloses the volume of interest.	$4\sqrt{\lambda_{major}}$
Minor Axis Length	Second largest axis length of the ellipsoid that encloses the volume of interest.	$4\sqrt{\lambda_{minor}}$
Least Axis Length	Smallest axis length of the ellipsoid that encloses the volume of interest.	$4\sqrt{\lambda_{least}}$
Elongation	Ratio of the two largest principal components of the volume of interest	$\sqrt{\frac{\lambda_{minor}}{\lambda_{major}}}$
Flatness	Ratio of the largest and smallest principal components of the volume of interest	$\sqrt{\frac{\lambda_{least}}{\lambda_{major}}}$

Column, M2DDR: Maximum2DDiameterRow, M2DDS: Maximum2DDiameterSlice, M3DD: Maximum3DDiameter, Volume: MeshVolume, SA: SurfaceArea, and SVR: SurfaceVolumeRatio.

Table 2: Morphological features of pre-operative computed tomography liver segmentation of 15 subjects.

Case	El.	Fl.	Sp.	MAL	MiAL	LAL	M2DDS	M2DDR	M2DDC	M3DD	Volume	SA	SVR
1	0.6317	0.4649	0.5490	223.0	140.9	103.7	243.0	189.7	222.8	265.5	1816935.0	131154.9	0.0722
2	0.5971	0.4260	0.4975	246.3	147.1	104.9	264.3	192.6	272.7	285.7	2027503.0	155700.6	0.0768
3	0.7173	0.5519	0.5835	205.4	147.3	113.4	215.5	199.1	236.2	236.8	1972033.0	130343.6	0.0661
4	0.8851	0.6374	0.5927	174.7	154.6	111.3	208.2	208.9	217.9	221.0	1800248.0	120745.0	0.0671
5	0.6275	0.5599	0.6215	230.4	144.6	129.0	263.1	202.7	241.5	273.8	2685685.0	150330.3	0.0560
6	0.6174	0.4145	0.5527	258.0	159.3	106.9	222.2	191.6	302.6	316.5	2458584.0	159379.2	0.0648
7	0.7791	0.4470	0.5430	205.5	160.1	91.9	240.5	201.2	232.8	241.8	1850338.0	134233.7	0.0725
8	0.7487	0.4433	0.5731	210.1	157.3	93.1	247.8	236.5	219.9	247.8	1809230.0	125298.7	0.0693
9	0.6209	0.4124	0.5579	219.4	136.2	90.5	225.8	184.9	239.9	263.8	1576820.0	117435.4	0.0745
10	0.6575	0.3935	0.5594	228.7	150.4	90.0	236.1	227.4	242.1	259.5	1916143.0	133362.1	0.0696
11	0.6580	0.4595	0.5273	233.8	153.8	107.4	283.5	200.7	292.8	297.9	1885451.0	139975.1	0.0742
12	0.6533	0.4662	0.5507	188.2	122.9	87.7	205.6	166.9	190.0	210.6	1105762.0	93901.1	0.0849
13	0.6315	0.4204	0.5561	201.4	127.2	84.7	204.3	183.5	194.9	223.2	1262017.0	101549.7	0.0805
14	0.5884	0.4167	0.5545	195.9	115.3	81.7	203.8	144.2	236.1	249.4	1083294.0	91991.8	0.0849
15	0.5755	0.4584	0.5661	208.4	119.9	95.5	207.6	164.9	216.7	235.6	1315777.0	102573.5	0.0780
Min	0.5755	0.3935	0.4975	174.7	115.3	81.7	203.8	144.2	190.0	210.6	1083294.0	91991.8	0.0560
Max	0.8851	0.6374	0.6215	258.0	160.1	129.0	283.5	236.5	302.6	316.5	2685685.0	159379.2	0.0849
Mean	0.6659	0.4648	0.5590	215.3	142.5	99.4	231.4	193.0	237.3	255.3	1771054.7	125865.0	0.0728
Median	0.6317	0.4470	0.5561	210.1	147.1	95.5	225.8	192.6	236.1	249.4	1816935.0	130343.6	0.0725

Table 3: Morphological features of registered to pre-operative CT, and then resampled intraoperative CT with pneumoperitoneum liver segmentation of 15 subjects.

Case	El.	Fl.	Sp.	MAL	MiAL	LAL	M2DDS	M2DDR	M2DDC	M3DD	Volume	SA	SVR
1	0.6773	0.4322	0.5073	220.5	149.4	95.3	243.0	175.3	226.0	269.6	1750489.0	138454.1	0.0791
2	0.5914	0.3801	0.4437	258.9	153.1	98.4	277.1	189.4	292.5	305.6	2121160.0	179915.4	0.0848
3	0.5694	0.3566	0.4860	237.0	135.0	84.5	239.9	166.7	240.0	258.9	1428120.0	126193.4	0.0884
4	0.7767	0.6025	0.5910	182.4	141.7	109.9	211.3	173.4	236.1	248.6	1686713.0	115940.8	0.0687
5	0.6795	0.5942	0.5672	216.2	146.9	128.5	226.0	197.4	259.4	275.3	2374794.0	151772.7	0.0639
6	0.7554	0.3883	0.4897	251.0	189.6	97.5	271.8	247.9	299.2	301.2	2456179.0	179782.1	0.0732
7	0.7381	0.3534	0.4823	211.9	156.4	74.9	242.1	188.8	221.9	244.6	1533381.0	133330.3	0.0870
8	0.7502	0.4191	0.5661	195.0	146.2	81.7	210.0	208.0	219.5	227.4	1463229.0	110106.4	0.0752
9	0.6180	0.3406	0.5050	222.0	137.2	75.6	249.6	168.5	216.3	253.9	1297187.0	113896.5	0.0878
10	0.5361	0.4816	0.5349	215.6	115.6	103.8	217.7	165.0	235.6	253.1	1488690.0	117864.9	0.0792
11	0.5480	0.4689	0.4965	239.2	131.1	112.2	277.1	169.1	310.5	313.7	1625524.0	134659.3	0.0828
12	0.7648	0.5189	0.5651	158.7	121.4	82.4	184.3	141.0	177.2	189.5	914399.6	80624.3	0.0882
13	0.5907	0.3583	0.5016	223.5	132.0	80.1	238.5	157.7	221.7	248.3	1269956.0	113065.6	0.0890
14	0.7203	0.5088	0.5380	166.8	120.1	84.9	201.9	159.5	220.6	228.2	965606.8	87818.4	0.0909
15	0.6272	0.5817	0.5233	174.1	109.2	101.3	198.7	142.7	189.3	212.4	1030344.0	94266.4	0.0915
Min	0.5361	0.3406	0.4437	158.7	109.2	74.9	184.3	141.0	177.2	189.5	914399.6	80624.3	0.0639
Max	0.7767	0.6025	0.5910	258.9	189.6	128.5	277.1	247.9	310.5	313.7	2456179.0	179915.4	0.0915
Mean	0.6629	0.4524	0.5198	211.5	139.0	94.1	232.6	176.7	237.7	255.3	1560384.8	125179.4	0.0820
Median	0.6773	0.4322	0.5073	216.2	137.2	95.3	238.5	169.1	226.0	253.1	1488690.0	117864.9	0.0848

Table 4: Morphological features of pre-operative computed tomography spleen segmentation of 15 subjects.

Case	El.	Fl.	Sp.	MAL	MiAL	LAL	M2DDS	M2DDR	M2DDC	M3DD	Volume	SA	SVR
1	0.6602	0.4492	0.6695	171.3	113.1	77.0	184.5	183.0	147.7	190.2	951912.5	69898.1	0.0734
2	0.7391	0.5211	0.6417	157.3	116.3	82.0	139.9	142.7	166.5	171.2	906510.7	70590.9	0.0779
3	0.7241	0.4474	0.7026	128.0	92.7	57.3	136.7	115.4	110.9	140.9	445849.7	40169.4	0.0901
4	0.7252	0.4590	0.6204	108.4	78.6	49.7	117.0	98.6	104.9	127.4	243127.2	30363.2	0.1249
5	0.6532	0.4070	0.6520	128.2	83.7	52.2	128.8	106.4	124.7	138.9	361241.5	37621.0	0.1041
6	0.7323	0.4486	0.6186	138.7	101.6	62.2	145.4	121.6	147.7	151.6	506681.8	49684.7	0.0981
7	0.6982	0.4383	0.5884	124.4	86.9	54.5	131.3	122.4	114.4	136.1	315887.5	38123.5	0.1207
8	0.7096	0.3856	0.6245	120.0	85.1	46.3	107.1	99.2	122.8	127.8	288239.9	33787.5	0.1172
9	0.6159	0.3202	0.5757	93.4	57.5	29.9	81.9	68.7	98.6	99.3	88539.8	16686.2	0.1885
10	0.7746	0.4144	0.6703	119.6	92.6	49.5	125.0	111.7	123.1	131.8	344845.6	35476.9	0.1029
11	0.5859	0.4462	0.6941	133.2	78.1	59.4	98.3	131.6	133.0	141.2	381533.3	36650.9	0.0961
12	0.6371	0.4060	0.5838	94.9	60.5	38.5	93.9	76.0	100.5	100.6	113754.7	19448.5	0.1710
13	0.6449	0.3539	0.6456	98.7	63.6	34.9	102.0	76.2	93.8	104.2	136475.7	19857.0	0.1455
14	0.5474	0.3730	0.6813	73.5	40.2	27.4	65.5	52.6	80.7	80.8	49802.1	9607.8	0.1929
15	0.6675	0.4615	0.6485	87.0	58.1	40.2	87.6	77.0	83.0	98.0	120861.0	18228.7	0.1508
Min	0.5474	0.3202	0.5757	73.5	40.2	27.4	65.5	52.6	80.7	80.8	49802.1	9607.8	0.0734
Max	0.7746	0.5211	0.7026	171.3	116.3	82.0	184.5	183.0	166.5	190.2	951912.5	70590.9	0.1929
Mean	0.6743	0.4221	0.6411	118.4	80.6	50.7	116.3	105.6	116.8	129.3	350350.9	35079.6	0.1236
Median	0.6675	0.4383	0.6456	120.0	83.7	49.7	117.0	106.4	114.4	131.8	315887.5	35476.9	0.1172

Table 5: Morphological features of registered to pre-operative CT, and then resampled intraoperative CT with pneumoperitoneum spleen segmentation of 15 subjects.

Case	El.	Fl.	Sp.	MAL	MiAL	LAL	M2DDS	M2DDR	M2DDC	M3DD	Volume	SA	SVR
1	0.6875	0.4362	0.6124	155.3	106.8	67.8	172.9	142.4	135.2	173.3	716529.0	63233.2	0.0882
2	0.6982	0.4874	0.5728	168.8	117.9	82.3	144.4	169.4	180.4	180.9	992271.6	83996.1	0.0847
3	0.7488	0.4260	0.6513	124.3	93.1	52.9	131.8	108.6	122.0	140.6	402439.7	40472.3	0.1006
4	0.8147	0.4707	0.5919	101.7	82.8	47.8	113.2	104.1	105.2	118.9	226784.7	30383.0	0.1340
5	0.6597	0.3922	0.6154	137.2	90.5	53.8	130.3	120.0	145.7	150.3	434407.6	45074.9	0.1038
6	0.7349	0.4359	0.5765	141.9	104.3	61.9	146.8	129.1	149.7	149.8	523131.5	54465.3	0.1041
7	0.6875	0.3924	0.5332	119.2	82.0	46.8	130.3	100.3	114.2	130.4	246788.4	35682.6	0.1446
8	0.6695	0.3885	0.6070	127.3	85.2	49.4	109.4	105.9	132.7	136.5	321978.7	37426.3	0.1162
9	0.5966	0.3020	0.5643	94.6	56.4	28.6	79.2	78.8	93.0	103.1	86760.4	16794.4	0.1936
10	0.7573	0.3736	0.6373	113.5	85.9	42.4	114.6	106.0	118.6	135.7	256738.2	30652.6	0.1194
11	0.5377	0.4096	0.6726	123.3	66.3	50.5	87.4	115.9	126.4	130.0	256766.9	29044.0	0.1131
12	0.6519	0.4322	0.5723	90.0	58.7	38.9	91.1	75.2	92.2	93.1	102813.4	18546.1	0.1804
13	0.5875	0.3344	0.6057	112.4	66.1	37.6	84.7	100.4	117.6	125.3	176673.8	25137.8	0.1423
14	0.5410	0.3524	0.6683	75.7	40.9	26.7	59.3	62.4	71.4	85.5	53220.1	10238.1	0.1924
15	0.6716	0.4404	0.6128	88.4	59.3	38.9	89.8	74.6	86.4	98.6	119899.0	19189.4	0.1600
Min	0.5377	0.3020	0.5332	75.7	40.9	26.7	59.3	62.4	71.4	85.5	53220.1	10238.1	0.0847
Max	0.8147	0.4874	0.6726	168.8	117.9	82.3	172.9	169.4	180.4	180.9	992271.6	83996.1	0.1936
Mean	0.6696	0.4049	0.6063	118.2	79.7	48.4	112.4	106.2	119.4	130.1	327813.5	36022.4	0.1318
Median	0.6716	0.4096	0.6070	119.2	82.8	47.8	113.2	105.9	118.6	130.4	256738.2	30652.6	0.1194

Table 6: Comparison of BMI (Body Mass Index), and volumes of pre- and intraoperative liver segmentations. CC-Disp.- Cranial-Caudal displacement. op.-operative. ⋆-underlying liver disease, ⋆⋆ represents minor shape alteration, †-liver cysts, ‡-pre-operative CT is CT colon, and intra CT image was taken after the liver tumor laparoscopic ablation, ⋄-Previous post-traumatic spleen.

Case	BMI	Liver status	Liver volume in (mL)		First liver slice in CT		CC-Disp.
			pre op.	intra op.	pre op.	intra op.	(in mm)
1	28.0	Cirrhosis, Steatosis	1816.9	1750.5	Th 10	Th 8	42
2	29.4	Cirrhosis	2027.5	2121.2	Th 9	Th 8	45
3	26.5	Steatosis, ⋆	1972.0	1428.1	Th 9	Th 8	18
4	28.1	Normal	1800.2	1686.7	Th 10	Th 8	48
5	31.5	Severe steatosis, ⋆⋆, †	2685.7	2374.8	Th 9	Th 6	69
6	35.1	Steatosis	2458.6	2456.2	Th 10	Th 8	48
7	26.0	Normal	1850.3	1533.4	Th 10	Th 8	78
8	24.5	Minor steatosis	1809.2	1463.2	Th 11	Th 10	36
9	26.3	Minor steatosis	1576.8	1297.2	Th 10	Th 9	40
10	26.3	Normal	1916.1	1488.7	Th 10	Th 8	45
11	28.1	Normal	1885.5	1625.5	Th 9	Th 7	47
12	17.4	Normal	1105.8	914.4	Th 11	Th 10	34
13	20.9	Steatosis, ‡	1262	1270	Th 10	Th 9	12
14	20.1	Normal, ⋄	1083.3	965.6	Th 12	Th 10	50
15	21.1	Normal	1315.8	1030.3	Th 11	Th 9	62
Min	17.4		1083.3	914.4			12
Max	35.1		2685.7	2456.2			78
Mean	25.9		1771.1	1560.4			45
Median	26.3		1816.9	1488.7			45
SD	4.6		453.6	468.7			17
